# Development and characterization of active packaging system based on zein nanofibers mat incorporated with geraniol‐loaded nanoliposomes

**DOI:** 10.1002/fsn3.4180

**Published:** 2024-04-22

**Authors:** Sara Gholizadeh, Hadi Almasi, Sajed Amjadi, Mehran Moradi, Nima Ghadiri Alamdari, Sorour Salmasi, Elahe Divsalar

**Affiliations:** ^1^ Department of Food Hygiene, Tabriz Branch Islamic Azad University Tabriz Iran; ^2^ Department of Food Science and Technology, Faculty of Agriculture Urmia University Urmia Iran; ^3^ Department of Food Nanotechnology Research Institute of Food Science and Technology (RIFST) Mashhad Iran; ^4^ Department of Food Hygiene and Quality Control, Faculty of Veterinary Medicine Urmia University Urmia Iran

**Keywords:** biodegradable packaging, controlled release, electrospinning, nanocarrier

## Abstract

In recent years, development of biopolymeric nanofibers as an active biodegradable packaging system has attracted specific attention. The objective of this research was to develop zein‐based electrospun nanofibers (NFs) incorporated with geraniol‐loaded nanoliposomes (G‐loaded NLPs). Geraniol was encapsulated into NLPs with an efficiency of 79.23%. The particle size and zeta potential of G‐loaded NLPs were 121.50 nm and −38.30 mV, respectively. The successful loading of geraniol in the NLPs was approved by Fourier transform infrared (FT‐IR) spectroscopy. The liposomal vesicles showed spherical shapes. G‐loaded NLPs were added in the zein‐based electrospun NFs at three different concentrations (0.25, 0.5, and 1%w/v). All NFs samples exhibited fibrillar structure. The increase of NLPs concentration enhanced the thermal stability of the NFs. However, the crystalline structure of zein NFs did not change by the addition of G‐loaded NLPs. The highest surface hydrophobicity was related to the NFs containing 1% G‐loaded NLPs. The mechanical parameters of NFs depend on the concentration of NLPs. The NFs incorporated with G‐loaded NLPs showed inhibition activity against four foodborne pathogenic bacteria (*Staphylococcus aureus*, *Listeria monocytogenes*, *Escherichia coli*, and *Salmonella typhimurium*) with an inhibition zone of 4.5–22 mm. Moreover, the α‐diphenyl‐β‐picrylhydrazyl (DPPH) scavenging activity of NFs samples was located at the range of 20%–48%. These findings represent the Efficiency of the G‐loaded NLPs for use as bioactive compound in the zein‐based NFs as an active packaging material.

## Graphical abstract

Geraniol (G) was successfully encapsulated in nanoliposome (NLPs) carriers and the active zein electrospun nanofibers were successfully reinforced with NPLs. All nanofibers were fibrillary in shape with relative crystalline nature and the G‐loaded NLPs created strong antioxidant/antimicrobial activity in fibers. The fabricated nanofibers are suggested as promising food active packaging.

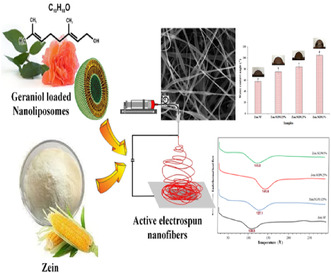

## 1 INTRODUCTION

In recent times, there has been a growing tendency toward the usage of novel food packaging techniques, caused by both consumer preferences and the industrial imperative to manufacture food products that are fresh, palatable, and easy to carry, while also possessing an optimal shelf‐life to ensure food quality (Marand et al., 2023; Shen et al., 2021).

Active food packaging technology is an innovative approach that aims to inhibit food contamination and ensure optimal food quality and safety. Furthermore, recent studies have indicated that the development of biodegradable biopolymeric packaging systems has been a response to the increasing environmental apprehension regarding the disposal of plastic packaging (Aman Mohammadi et al., 2021). The use of biodegradable films and nanofibers (NFs) as active food packaging systems is a promising and intriguing approach in the food industry for improving the safety and extending the shelf‐life of food products (Ma et al., 2021).

Electrospinning as a technique for the production of nanofibers (NFs) has gained significant attention in the food packaging field (Yu et al., 2023). So that, this technique has several advantages, including a cost‐effective and straightforward approach as well as its ability to generate three‐dimensional (3D) NFs structures with desirable properties, such as high porosity and continuity (Wahbi et al., 2020). Furthermore, the electrospun NFs provide controlled release profile for loaded active compounds in the NFs structure (Ma et al., 2023). This characteristic is an advantage for an active packaging system, as it prolongs the shelf‐life of foodstuffs (Ahmadi et al., 2024).

Zein is a macromolecular protein with nonpolar amino acids that provide hydrophobic characteristics and is frequently utilized as a biopolymer in the production of NFs. The biocompatibility, nontoxicity, glossiness, tensile strength, and hydrophobicity of this biopolymer make it a desirable material for this purpose (Amjadi, Gholizadeh, et al., 2022c; Bayer, 2021; Jiang et al., 2022). Recently, zein‐based electropun NFs have been used for packaging of different food products, such as pork (Xia et al., 2023), strawberries (Wu et al., 2023), sweet potatoes, potatoes, kimchi (Ullah et al., 2023), and fresh rainbow trout fillet (Ahmadi et al., 2024). The results of these studies showed the potential of the zein‐based NFs for extending the shelf‐life of different foodstuffs.

Meanwhile, attempts are being made to enhance the functionality of packaging materials through the incorporation of antimicrobial and antioxidant agents. Active packaging materials are defined as those that contain components with biological characteristics that are gradually released into the food product.

Geraniol (C_10_H_18_O) is an aliphatic monoterpene with a functional alcohol group as its essential organic structure (Lira et al., 2020). This particular compound is classified as a secondary metabolite and has been identified in various plant species, such as lemongrass, rose, and citrus (Agarwal et al., 2020). Geraniol is recognized for its pharmacological properties and is considered a potent agent for anthelmintic, anti‐inflammatory, anticancer, and antioxidative purposes (Chatterjee et al., 2022; Wróblewska et al., 2022). The investigation of nanoparticles that contain geraniol and their effectiveness in combating *Klebsiella, Escherichia coli*, and *Staphylococcus aureus*, as well as their ability to inhibit biofilm formation in *Staphylococcus epidermidis*, offers new perspectives and reinforces the potential of geraniol as an active packaging component (Wróblewska et al., 2022). Notwithstanding the aforementioned advantages, the sustainability and hydrophobicity of geraniol present significant obstacles due to their volatile properties, which impact their efficacy (Carniel et al., 2021).

Encapsulation with nanocarriers, particularly those based on lipids, can enhance the stability of bioactive compounds in the face of environmental factors, thereby addressing the drawbacks mentioned earlier. Nanoliposomes (NLPs) are a type of lipid‐based nanocarriers that offer the advantage of encapsulating both hydrophilic and hydrophobic ingredients due to their diverse structural composition. NLPs exhibit several advantageous characteristics, including biocompatibility, amphiphilicity, and the ability to improve the bioaccessibility of loaded active compounds, owing to their nanoscale dimensions (Marand et al., 2023; Tavares et al., 2021).

In recent years, some studies have been performed about the incorporation of antibacterial compounds, such as thymoquinone (Ahmadi et al., 2024), piperine (Daneshmand et al., 2023), cinnamaldehyde/thymol (X. Wu et al., 2023), peppermint oil (Xia et al., 2023), nisin (Yu et al., 2023), and curcumin (Li et al., 2024), in zein‐based NFs. However, no research has been conducted on the use of geraniol as an active agent in packaging systems, especially NFs. Besides, there is no research on the preparation of NLPs incorporated in zein NFs. Therefore, the present study aimed to (a) encapsulate geraniol by NLPs and (b) prepare the zein electrospun NFs incorporated with geraniol NLPs. The ultimate goal of this work was to develop an active biopolymeric packaging system to extend the shelf‐life of different kinds of food products.

## 2 MATERIALS AND METHODS

### 2.1 Materials

Geraniol (99.9% purity), soybean lecithin, Zein (99.5% purity, grade Z3625, 22–24 kDa), α‐diphenyl‐β‐picrylhydrazyl (DPPH), and other chemical ingredients were obtained from Sigma‐Aldrich (USA). Plate count agar (PCA) was purchased from Quelab (Canada).

### 2.2 Preparation of geraniol‐loaded NLPs

Initially, a solution comprising 90 mg of lecithin and 20 mL geraniol was dissolved in 15 mL ethanol, and subsequently, the resultant solution was transferred into a round bottom flask. Then, the ethanol was evaporated using a rotary evaporator (Laborota 4002, Heidolph, Germany), forming a thin film around the flask. The lipid film obtained was hydrated with 10 mL of deionized water. The liposomal dispersion was then homogenized (Silent Crusher M, Heidolph, Germany) for 20 min at 20,000 rpm (revolutions per minute) and 60°C. Subsequently, the liposomal sample was subjected to sonication using a probe ultrasound sonicator (UP200H, Hielsher, Germany) for a total of 10 cycles, with each cycle consisting of 1 min of sonication followed by 1 min of rest, at a sonication power of 70% (Amjadi et al., 2023).

### 2.3 Characterization of geraniol‐loaded NLPs

#### 2.3.1 Zeta potential, particle size, and PDI measurement

For the purpose of determining the zeta potential, mean particle size, and polydispersity index (PDI) of NLPs, a photon correlation spectroscopy (PCS) (Zetasizer Nano‐ZS, Malvern, UK) was utilized.

#### 2.3.2 Encapsulation efficiency (EE) measurement

The diluted samples of liposome were transferred to an Amicon® filter (molecular weight cutoff 100 kDa, Millipore, UK) and centrifugation was done at 3000 rpm for 20 min. The absorbance of the solution that passed through the filter (containing unloaded geraniol) was measured by using a Ultraviolet–Visible (UV–Vis) spectrophotometer (Ultrospec 2000, Pharmacia Biotech, Cambridge, UK). The concentration of geraniol was determined from its calibration curve. The following equation was used to calculate the EE value of geraniol (Ebrahimi et al., 2023):

(1) $$\text{Encapsulation efficiency}\%=\frac{\text{Total compound}\;\left(w\right)-\text{unloaded compound}\;\left(w\right)}{\text{Total compound}\;\left(w\right)}\times 100$$

#### 2.3.3 Morphology study

Utilizing field emission scanning electron microscopy (FE‐SEM) (MIRA3, TESCAN, Brno, Czech Republic), the morphology of nanoliposome samples was analyzed. The diluted samples at a ratio of 1:10 were placed on glass slides and desiccated at 25°C, prior to the test. Under a vacuum, the samples were then coated with a thin layer of gold using the direct current sputtering technique (DST1, Nanostructured Coating Co., Tehran, Iran). The accelerating voltage applied was 15 kV.

#### 2.3.4 Chemical structure study

Using Fourier transform infrared red (FT‐IR) spectroscopy (Equinox 55 LS 101, Bruker, Karlsruhe, Germany), the interactions between various components of NLPs samples were evaluated. Potassium bromide (KBr) pellets were used to embed the samples. The 400–4000 cm^−1^ wavenumber range with set resolution of 2 cm^−1^ was used to record the spectra.

### 2.4 Production of electrospun NFs

First, zein powder at a ratio of 20% (w/v) was dissolved in the ethanol (80% v/v) at 25°C. Subsequently, geraniol‐loaded (G‐loaded) NLPs were added to the polymer solution at concentrations of 0.25, 0.5, and 1% w/v. After that, the mixtures were stirred at 25°C for 12 h. The prepared solutions were loaded into a 5 mL plastic syringe with a 21‐gauge stainless steel needle for the electrospinning procedure; 17 kV and 0.5 mL/h were set as the applied voltage and flow rate, respectively. NFs were captured at a horizontal distance of 10 cm from the needle tip on an aluminum foil collector. The electrospinning procedure was carried out at a temperature of 25 ± 2°C and a relative humidity of 30 ± 1% (Amjadi et al., 2020a).

### 2.5 Characterization of NFs

#### 2.5.1 Scanning electron microscopy (SEM)

Field emission scanning electron microscopy (FE‐SEM) was applied to observe the morphology of NFs samples that had been prepared. Prior to conducting the analysis, the samples underwent a process in which they were coated with a thin layer of gold. The distribution of fiber diameters was estimated by measuring 100 randomly chosen spots in FE‐SEM images with ImageJ software.

#### 2.5.2 Differential scanning calorimetry analysis

The thermal behavior of nanofiber samples was investigated using differential scanning calorimetry (DSC) (PerkinElmer Thermal Analysis, Germany). Initially, a quantity of 6 mg of samples was carefully deposited into a sealed aluminum pan. Subsequently, the samples that had been prepared were subjected to a controlled heating process, wherein the temperature was increased from 25°C to 300°C at a rate of 10°C/min, all while maintaining a nitrogen atmosphere.

#### 2.5.3 X‐ray diffraction (XRD) analyses

X‐ray diffraction (XRD) analyses were conducted on the NFs utilizing a Bruker D8 Advance X‐ray diffractometer (Karlsruhe, Germany). This instrument was employed to examine the crystalline arrangement of the NFs within the diffraction angle (2θ) range of 10–60°. The CuKα emission at 0.1564 nm was utilized, and the scanning rate was set at 1°/min at 25°C.

#### 2.5.4 Fourier transform infrared (FT‐IR)

The assessment of interactions among different components in the NFs was conducted through the utilization of FT‐IR spectroscopy according to the procedure mentioned in Section 2.3.4.

#### 2.5.5 Water contact angle

In order to determine the water contact angles of NFs, a volume of 5 μL of distilled water was dropped onto the nanofiber surface until a stable water contact angle was achieved. Subsequently, the nanofiber samples were imaged utilizing a camera (Microsoft, LifeCam, H5D‐00013, zoom 24×). The contact angles exhibited by the water droplets were determined using ImageJ software (Marand et al., 2023).

#### 2.5.6 Mechanical properties

Using an Instron 5566 Tensile Analyzer (USA), the tensile properties of film samples were analyzed. The films were first cut into dumbbell‐shaped segments measuring 5 × 1 cm^2^. At 25°C, the initial gap between the two grips was 50 mm, and the speed was 10 mm/min. The analysis produced three parameters: Young's modulus (YM), elongation at break (EAB), and ultimate tensile strength (UTS) (Yu et al., 2023).

#### 2.5.7 Antimicrobial activity

The disk diffusion method was used to assess the antibacterial activity tests against *Staphylococcus aureus* (ATCC®12600™), *Listeria monocytogenes* (ATCC®7644™), *E. coli* (ATCC®11775™), and *Salmonella typhimurium* (ATCC®14028™) bacteria (Amjadi, Gholizadeh, et al., 2022c).

#### 2.5.8 Antioxidant activity

The DPPH assay was applied for measuring the antioxidant axtivity of NFs (Amjadi, Almasi, et al., 2022a). Following that, 0.2 mL of DPPH ethanolic solutions (0.04 g/L) was mixed with 1 mL of NFs extract solution and the mixture was maintained at 25°C in a darkroom for 30 min. Following this, a cuvette was filled with 1 mL of each solution obtained, and their absorbance at a wavelength of 517 nm was measured using a UV–Vis spectrophotometer (Ultrospec 2000, Biotech, UK). In conclusion, the DPPH free radical scavenging activity was determined using the equation provided below:

(2) $$\text{Free radical scavenging activity}\;\left(\%\right)=\frac{{\mathrm{Abs}}_{\text{control}}-{\mathrm{Abs}}_{\text{sample}}\;}{{\mathrm{Abs}}_{\text{control}}}\times 100$$

where Abs_control_ and Abs_sample_ represent the absorbance of the blank and the absorbance of the sample at 517 nm, respectively.

### 2.6 Statistical analysis

The sampling method was random and all tests were performed in triplicate. One‐way analysis of variance (ANOVA) was conducted by IBM SPSS Statistics 21 (Version 23, SPSS Inc., Chicago, IL, USA) for statistical analysis of data. Following, Duncan's mean comparison test was conducted at 5% significant level.

## 3 RESULTS AND DISCUSSION

### 3.1 Characterization of geraniol‐loaded NLPs

#### 3.1.1 Particle size and zeta potential measurement

The particle size, zeta potential, and PDI of free and G‐loaded NLPs are displayed in Table 1. It is established that the size of NLPs was significantly impacted (*p* < .05) by the loading of geraniol. The NLPs' diameter was initially measured at 98.05 ± 3.21 nm, however, subsequent to the loading of geraniol, the size of the NLPs exhibited an increase to 121.50 ± 4.21 nm. The observed augmentation in particle size is a typical phenomenon that signifies the efficacious incorporation of geraniol within the structure of NLPs. The average size of NLPs that encapsulated geraniol was found to be within the optimal range, suggesting the potential applicability of this nanocarrier system in the next stages of research. So that, the obtained particle size was smaller than the reported particle size for thymol‐ and carvacrol‐loaded NLPs (Heckler et al., 2020) and ginger essential oil‐loaded NLPs (Ekrami et al., 2023) that their average particle sizes were 230 and 154 nm, respectively.

**TABLE 1:** Particle size, zeta potential, and PDI of free and G‐loaded NLPs.

Sample	Particle size (nm)	PDI	Zeta potential (mV)
Blank NLP	98.05 ± 3.21^a^	0.40 ± 0.07^a^	−40.70 ± 2.11^a^
G‐loaded NLP	121.50 ± 4.21^b^	0.45 ± 0.05^b^	−38.30 ± 3.00^a^

*Note*: Different letters show significant differences at the 5% level in Duncan's test (*p* < .05).

Abbreviations: *G*, geraniol; NLPs, nanoliposomes.

The PDI index of the NLPs produced by the thin‐layer hydration method in this study was 0.4, which is comparable to the results obtained by Rafiee et al. (2017) regarding the NLPs produced by the same method and containing pistachio green skin extract. The PDI value of G‐loaded NLPs showed no significant difference (*p* > .05) in comparison with the PDI value of blank NLPs. This shows that the geraniol does not have a negative effect on the production conditions of the nanoliposome due to its low molecular weight and the ability to completely cover the hydrophobic layer of the nanoliposome and does not interfere with the production conditions. In general, the PDI results were found to be within the acceptable range and confirmed the production of NLPs with a narrow particle size distribution range.

As shown in Table 1, the zeta potential for blank NLPs was −40.70 ± 2.11 mV. This negative charge is due to the anionic phosphate groups in the polar part of the lecithin structure, which is placed in the surface layer of NLPs and causes this negative charge. A similar result has been observed by previous studies (Haghju et al., 2016; Wu et al., 2015). By adding geraniol, there was no significant change in the amount of zeta potential (*p* < .05). The slight decrease in the zeta potential to −38.30 ± 3.00 mV can be attributed to the presence of uncoated geraniol on the surface of the NLPs and its binding with phosphate groups, and as a result, the reduction of the negative charge of the NLPs. However, the values of zeta potential in the NLPs produced were more than 30 mV, which indicates the favorable electrostatic stability of G‐loaded NLPs.

#### 3.1.2 Encapsulation efficiency (EE) measurement

The amount of the desired substances loaded inside the nanocarriers is represented by EE. The most important variables influencing the EE of NLPs are the nature of the loaded materials, including their hydrophilicity or hydrophobicity, the stabilizers used, and the production technique. In this research, the EE of geraniol was 79.23 ± 4.20%. The placement of geraniol within the NLPs is attributed to its hydrophobic properties, which allow it to reside within the interstitial space between the hydrophobic layers rather than the central hydrophilic core. The numerical value obtained for this encapsulation process is deemed satisfactory for a hydrophobic substance within the NLPs. This value is more than the EE reported for curcumin (68.3%) (Chen et al., 2015) and cinnamon essential oil (72.6%) (Wu et al., 2015) encapsulation in NLPs produced by the thin‐layer hydration method, which shows the acceptable efficiency of the conditions used in this research.

#### 3.1.3 Chemical structure study (FT‐IR)

**FIGURE 1:**
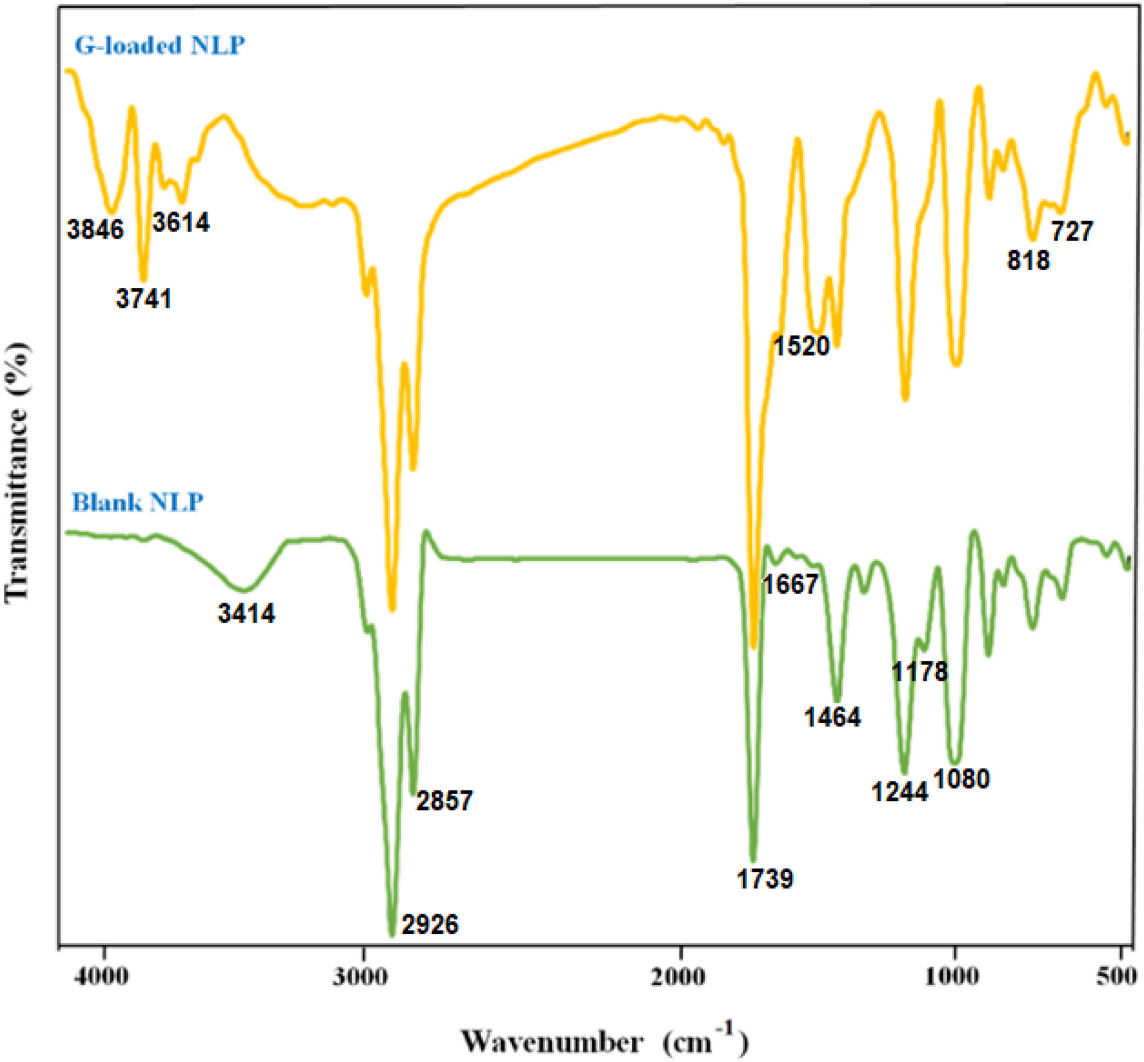
The Fourier transform infrared (FT‐IR) spectra of the blank and G‐loaded NLPs. *G*, geraniol; NLPs, nanoliposomes.

The FT‐IR spectra of the blank and G‐loaded NLPs are demonstrated in Figure 1. The distinct peaks of the blank NLP are found to be located at 3414 cm^−1^ for O–H stretching vibrations, 2926 and 2857 cm^−1^ for C–H stretching vibrations, 1739 and 1667 cm^−1^ for C=O groups, 1464 cm^−1^ for C–H bands, 1244 cm^−1^ for C–N stretching of the amine group, and 1178 and 1080 cm^−1^ for asymmetric and symmetric stretching vibrations of PO_2_ groups, respectively (Amjadi, Almasi, et al., 2022a).

The loading of geraniol in the NLPs resulted in some spectral alterations, such as the disappearance of the peak at 3414 cm^−1^ and the appearance of new peaks at higher wavenumbers (3614, 3741, and 3846 cm^−1^), which are associated with the benzene ring group of the geraniol. In addition, the peak at 1464 cm^−1^ was shifted to a higher wavenumber of 1520 cm^−1^. Geraniol loading also enhanced the intensity of peaks between 600 and 800 cm^−1^. These changes indicate the establishment of new chemical connections between phospholipids and geraniol. Similar results have been reported regarding stabilizing curcumin (Chen et al., 2015) and betanin and carvone (Amjadi, Almasi, et al., 2022a, 2022b) in NLPs produced by the same method. But, Mohammadi et al. (2014) did not confirm any interaction between encapsulated nutraceutical and liposome constituents in the coating of vitamin D_3_ in NLPs.

#### 3.1.4 Morphology study

**FIGURE 2:**
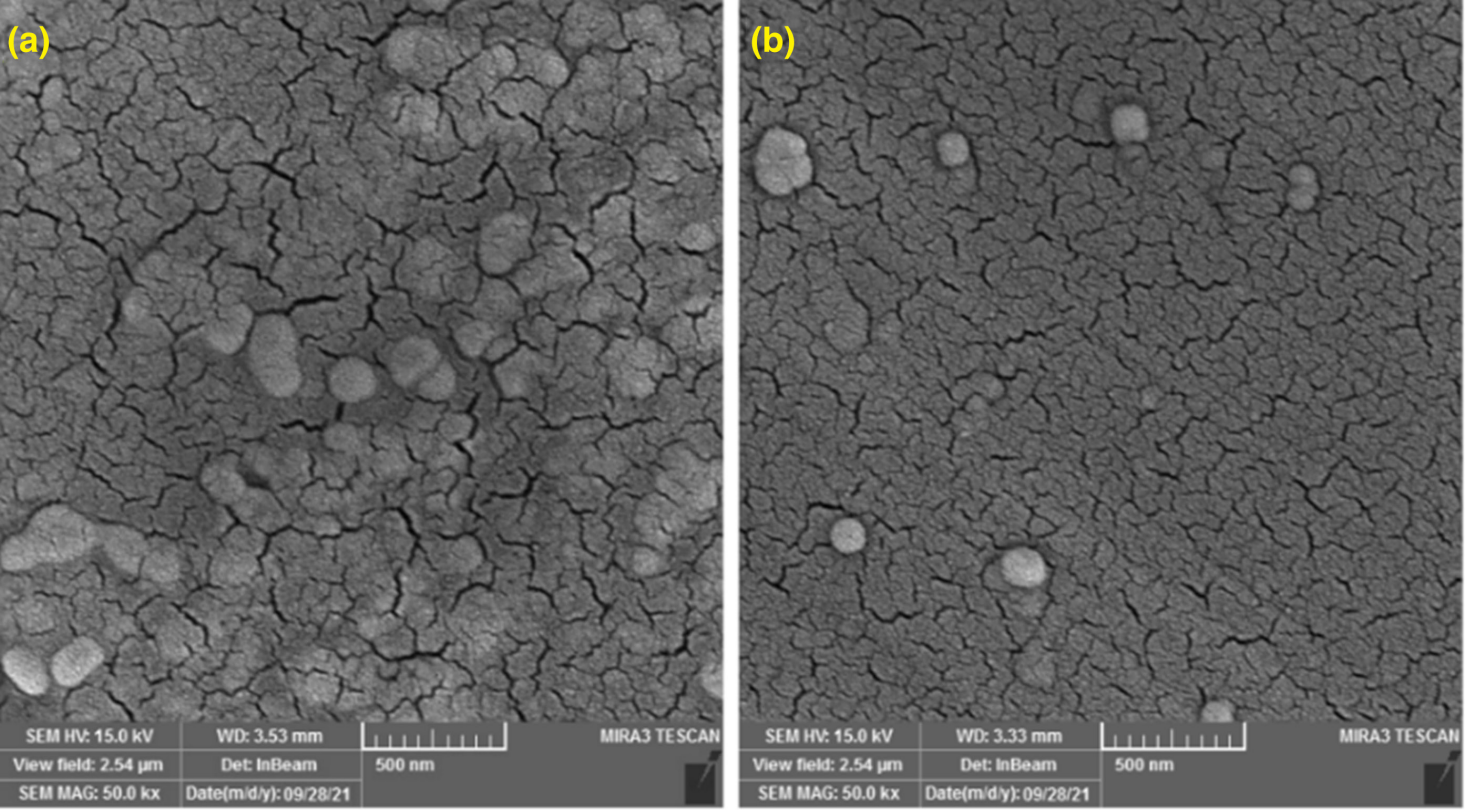
Scanning electron microscopy (SEM) images of the blank (a) and G‐loaded (b) NLPs. G, geraniol; NLPs, nanoliposomes.

Figure 2 depicts the FE‐SEM images of the blank (A) and G‐loaded (B) NLP samples. It is obvious that the morphology of the NLPs remains spherical both in the blank state and subsequent to geraniol loading. The addition of geraniol did not result in a significant alteration of NLPs morphology. The only observable change was the reduction in clumping and nonaggregation of NLP particles after geraniol loading. The partial presence of geraniol molecules on the surface of NLPs, which decreases surface hydrophilicity and restricts the tendency of particles to aggregate, is likely responsible for this outcome. In general, the SEM test results confirmed the efficacy of the G‐loaded NLPs production.

Following the successful synthesis of NLPs that incorporate geraniol, the next step involved utilizing this nanosystem in the method of electrospinning in order to produce zein NFs. The outcomes of this experiment are detailed below.

### 3.2 Characterization of NFs

#### 3.2.1 SEM

**FIGURE 3:**
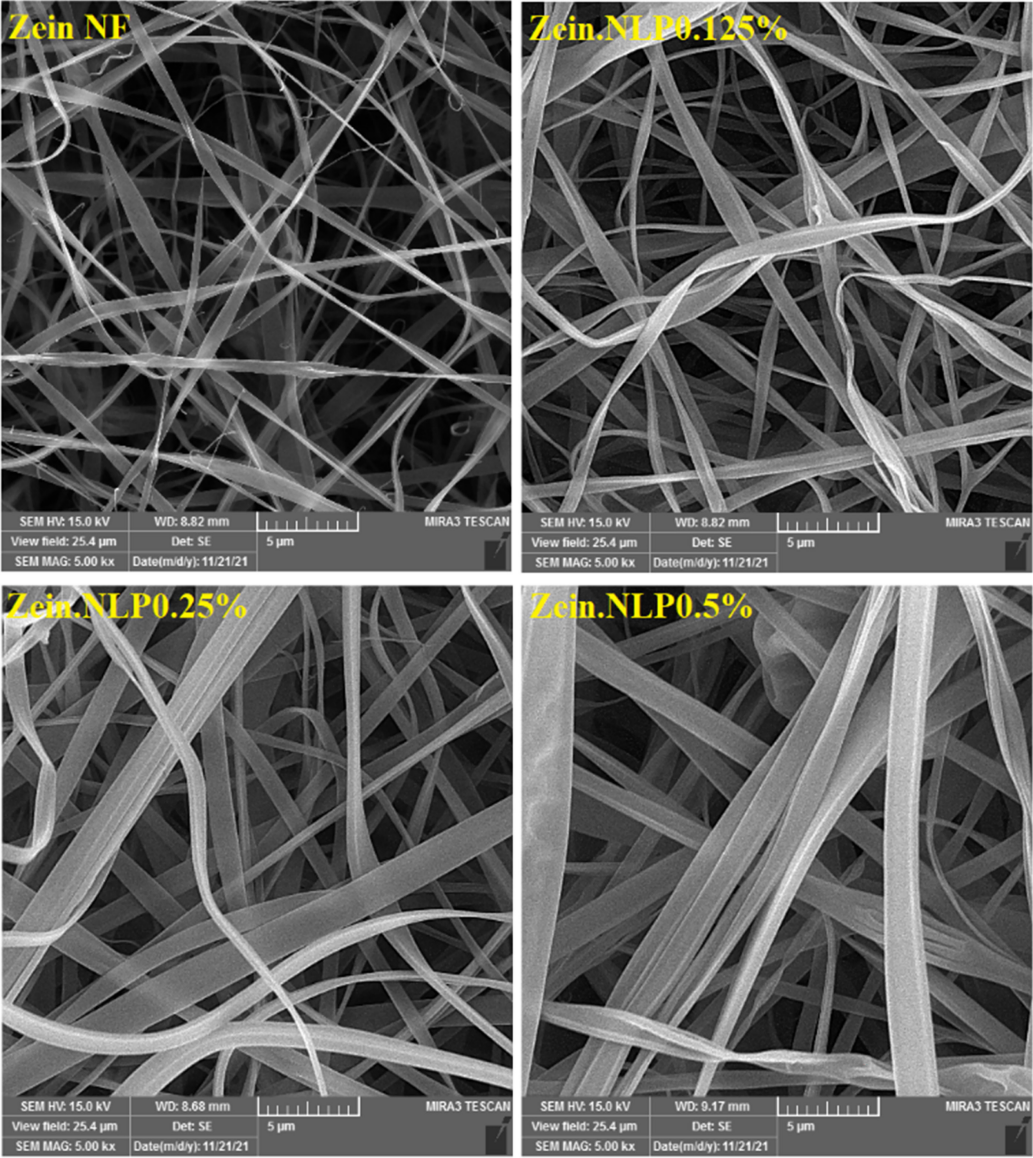
Field emission scanning electron microscopy (FE‐SEM) images of neat zein, zein.NLP 0.125%, zein.NLP 0.25%, and zein.NLP 0.5% NFs. NFs, nanofibers; NLP, nanoliposome.

As seen in Figure 3, all the NFs produced in this research had a ribbon‐like morphology that was a commonly observed morphology for zein‐based NFs (Alehosseini et al., 2019; Altan et al., 2018). The collapse of NFs can be caused by the rapid vaporization of ethanol from the jet surface. This is while Moreno et al. (2019) observed an ultrathin fibrillar structure with a cylindrical shape in the images of pure zein NFs. Additionally, the average diameter of neat zein NFs was 384 nm. By the addition of G‐loaded NLPs to the zein‐based NFs at different concentrations, no significant changes were seen in the morphology of the NFs, but the fiber diameter was increased (*p* < .05). The SEM image of zein.NLP 0125% nanofiber sample exhibited that the average diameter reached 437 nm, but this difference increased at higher concentrations. The average diameter of zein.NLP 0.25% nanofiber sample was 955 nm, and in the sample with the highest amount of NLPs, it reached 1022 nm with a slight increase. All samples maintained the smooth ribbon shape of the NFs, but the diameter of the ribbon increased. The effect of incorporation of free and encapsulated bioactive compounds on increasing the diameter of electrospun zein NFs has also been proven in previous reports (Altan et al., 2018; Amjadi et al., 2020a, 2020b). Another important point that is evident in the FE‐SEM images is that despite the increase in the diameter of the NFs, there is no change in their uniformity, and no beaded regions or other nonuniform points are observed in the morphology of the zein NFs containing NLPs. This shows the good compatibility of NLPs with zein protein, which is also confirmed in FT‐IR results.

#### 3.2.2 DSC analysis

**FIGURE 4:**
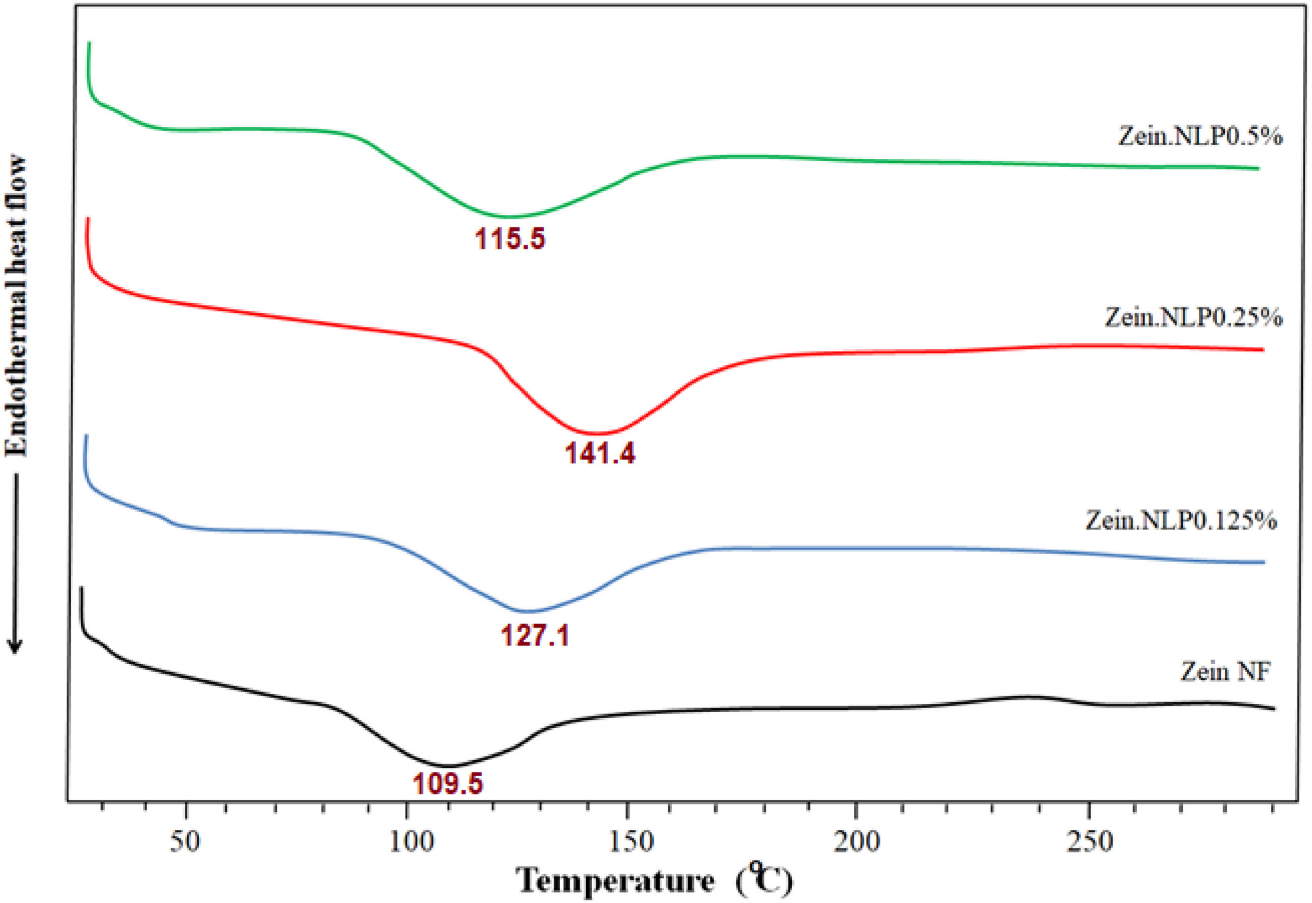
Differential scanning calorimetry (DSC) thermograms of neat zein, zein.NLP 0.125%, zein.NLP 0.25%, and zein.NLP 0.5% NFs. NFs, nanofibers; NLP, nanoliposome.

Figure 4 depicts the DSC thermograms of the samples. No sharp endothermic peak was seen in the thermogram of control sample because of the amorphous structure of zein (Lu et al., 2017). The glass transition temperature (*T*_g_) of pure zein NFs was 109.5°C. This temperature is consistent with Shen et al. (2021)'s report from (118°C). However, it is greater than the *T*_g_ (69.7°C) found for zein NFs in the research of Deng et al. (2019). These variations demonstrate that the NFs' production conditions, dry matter content, fiber diameter, and zein chain density in their structural makeup all have an impact on their thermal behavior. As a result, a wide range of *T*_g_ have been observed in various studies.

The addition of G‐loaded NLPs resulted in an initial increase in the *T*_g_. The temperature at which the lowest concentration of NLPs appeared was measured to be 127.1°C. In contrast, the sample containing 0.25% NLPs exhibited the highest *T*_g_, which reached a maximum value of 141.4°C. By intercalating NLPs within the polymer chains and facilitating the formation of new chemical linkages, the overall structural organization is enhanced, leading to a decrease in the mobility of the chains. The reduction in molecular mobility leads to an increase in the *T*_g_ of NFs. Kayaci and Uyar (2012) observed that by incorporating cyclodextrins into the structure of electrospun zein NFs, the *T*_g_ of zein NFs increased due to the formation of new interactions between zein and cyclodextrins. de Freitas Zômpero et al. (2015) also reported that the addition of NLPs containing beta‐carotene improved the thermal characteristics of polyvinyl alcohol and polyethylene oxide NFs. Bioactive compounds with the capacity to form chemical bonds with NFs can aid in increasing *T*_g_. Similar results have been reported for the effect of betanin (Amjadi et al., 2020b), vanillin (Drago et al., 2022), cinnamic aldehyde (Karim et al., 2021), and resveratrol (Leena et al., 2020) on increasing the *T*_g_ of zein NFs. Figure 4 demonstrates that by increasing the concentration of NLPs to 0.5%, the *T*_g_ of NFs decreased to 115.5°C. Probably, the nongrafted NLPs and the free and nonencapsulated geraniol function as softeners and prevent the zein chains from approaching and forming a regular arrangement, resulting in a decrease in the *T*_g_ of active NFs due to an increase in molecular mobility.

#### 3.2.3 XRD analyses

**FIGURE 5:**
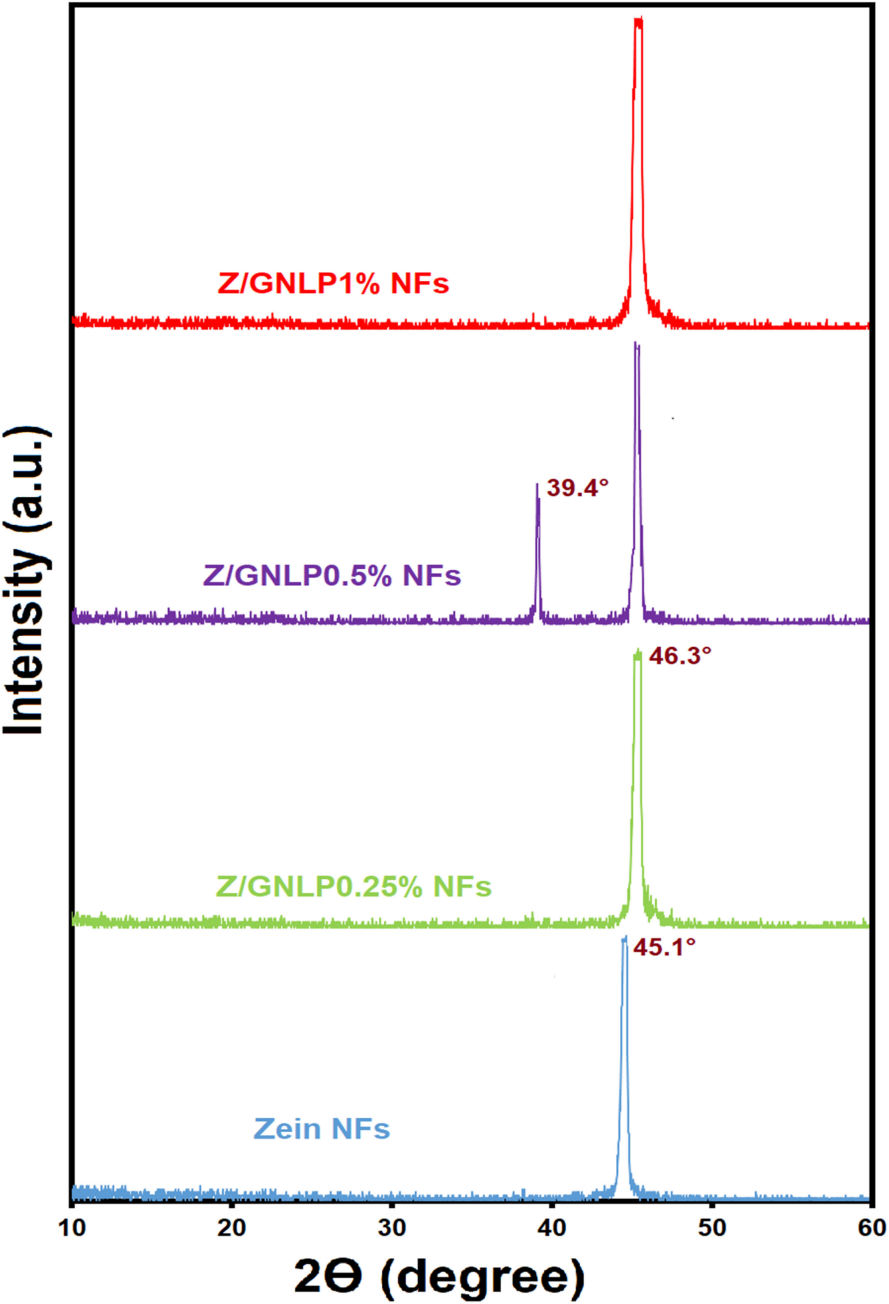
X‐ray diffraction (XRD) patterns of neat zein, Z/GNLP 0.25%, Z/GNLP 0.5%, and Z/GNLP 1% NFs. GNLP, geraniol‐loaded NLP; NFs, nanofibers; NLP, nanoliposome; Z, zein.

Figure 5 presents the internal structure and crystallinity characteristics of zein NFs with and without G‐loaded NLPs. Neat zein nanofibers exhibited a distinct peak at 2θ of 45.1°. The obtained outcome demonstrates that zein NFs, produced through the process of electrospinning, reveal characteristics of a semicrystalline substance. This observation aligns with the conclusions drawn by a previous study (Amjadi, Gholizadeh, et al., 2022c) in their investigation of zein NFs. Following the addition of G‐loaded NLPs, no discernible changes in the intensity of the zein peak were detected, but a small shift was made in its location and moved to 2θ of 46.3°. This demonstrates that while the addition of NLPs has no impact on the crystallinity of zein NFs, the development of novel contacts following the addition of NLPs results in a change in the crystal's form and morphology, which alters the crystal peak's location. Similar changes in the crystallinity properties of the films of pectin and isolated whey protein have been observed with the addition of marjoram essential oil in free and encapsulated form (Almasi et al., 2020) and black cumin essential oil in free and encapsulated form (Hemmatkhah et al., 2020). The new peak appearing in the sample containing 0.5% NLPs is probably due to the presence of impurities in this sample. In another study (Aziz & Almasi, 2018), it has been found that the addition of thyme extract‐loaded NLPs did not adversely impact the crystalline structure of whey protein‐based films. Researchers attributed this phenomenon to the presence of double amphiphilic lecithin molecules within the NLPs, which facilitated increased interactions with the hydrophilic protein matrix.

#### 3.2.4 FT‐IR analyses

**FIGURE 6:**
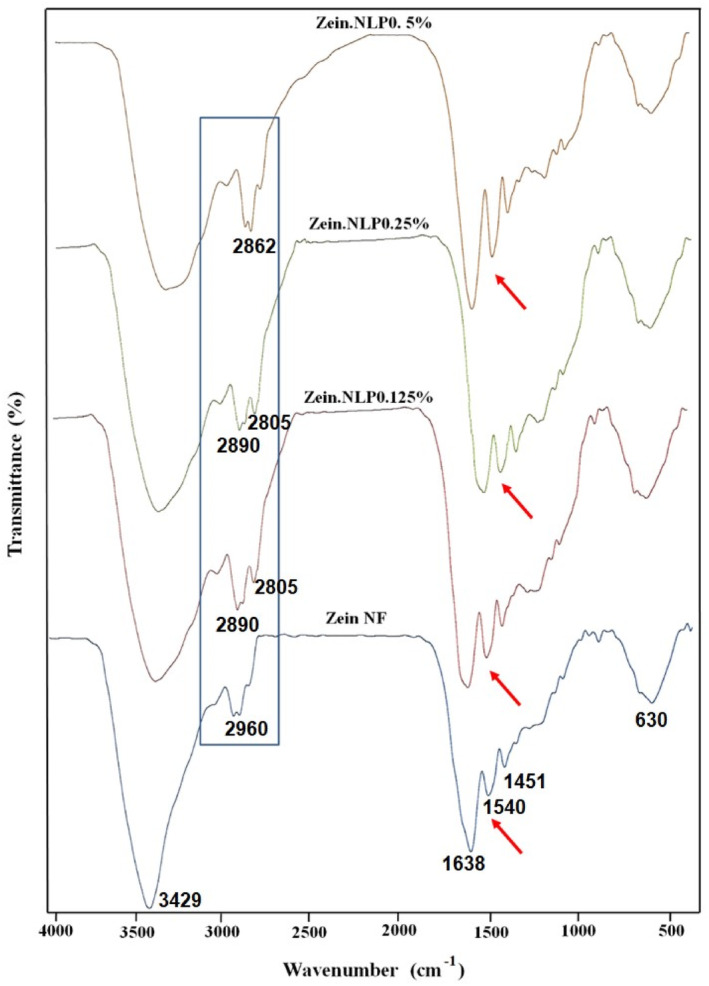
Fourier transform infrared (FT‐IR) spectra of neat zein, zein.NLP 0.125%, zein.NLP 0.25%, and zein.NLP 0.5% NFs. NFs, nanofibers; NLP, nanoliposome.

The FT‐IR test was employed to examine the chemical structure of zein and NLPs, as well as to explore the potential for establishing new interactions between them. Figure 6 presents the FT‐IR spectra of neat zein, zein.NLP 0.125%, zein.NLP 0.25%, and zein.NLP 0.5% nanofiber samples. The spectrum of control NFs exhibited several notable peaks, including a) the peak at 3429 cm^−1^, which represented the O–H stretching of the amino acids; b) a peak at 2960 cm^−1^, corresponding to the C–H stretching vibrations; c) the peaks at 1638 cm^−1^, 1540 cm^−1^, and 1451 cm^−1^, which indicated the strong absorption bonds of amide I or imine, amide II, and amide III, respectively; and d) a peak at 630 cm^−1^, which was associated with the O–H stretching of carboxylic acids (Deng et al., 2019). The obtained results exhibited congruence with the research outcomes reported by Wang et al. (2019) and Amjadi et al. (2020b). As shown in Figure 6, spectral changes were observed by the addition of G‐loaded NLPs in the zein‐based NFs at different concentrations. An important change is the shift of the 2960 cm^−1^ peak to a lower wavenumber. In the samples containing 0.125% and 0.25% of NLPs, the position of this peak decreased to 2890 cm^−1,^ and a new peak was created next to the main peak (2805 cm^−1^). In the sample containing 0.5% NLPs, this peak was shifted again and moved to 2862 cm^−1^. Another notable distinction observed was the alteration in both the intensity and peak position of the amide groups. In the sample with a concentration of 0.125% NLPs, a reduction in the intensity of these peaks was observed. In the sample containing 0.25 NLPs, there was a reduction in peak intensity accompanied by a slight shift in peak position. Subsequently, in the sample with the highest nanoliposome concentration, the peak intensity increased, and the peaks returned to their original position. The addition of NLPs to the zein protein results in notable alterations in the intensity and position of the protein's peaks. These changes reflect the impact of the nanocarrier on the chemical composition and the formation of novel chemical bonds between NFs and NLPs. In previous studies, the establishment of chemical interactions and the formation of hydrogen bonds between beta‐carotene NLPs and polyvinyl alcohol and polyethylene oxide NFs were reported by de Freitas Zômpero et al. (2015). Similarly, the formation of betanin NLPs and gelatin/chitosan NFs was demonstrated by Amjadi et al. (2020b). Additionally, the successful creation of tea tree liposome and chitosan NFs was confirmed by Cui et al. (2018).

#### 3.2.5 Water contact angle

**FIGURE 7:**
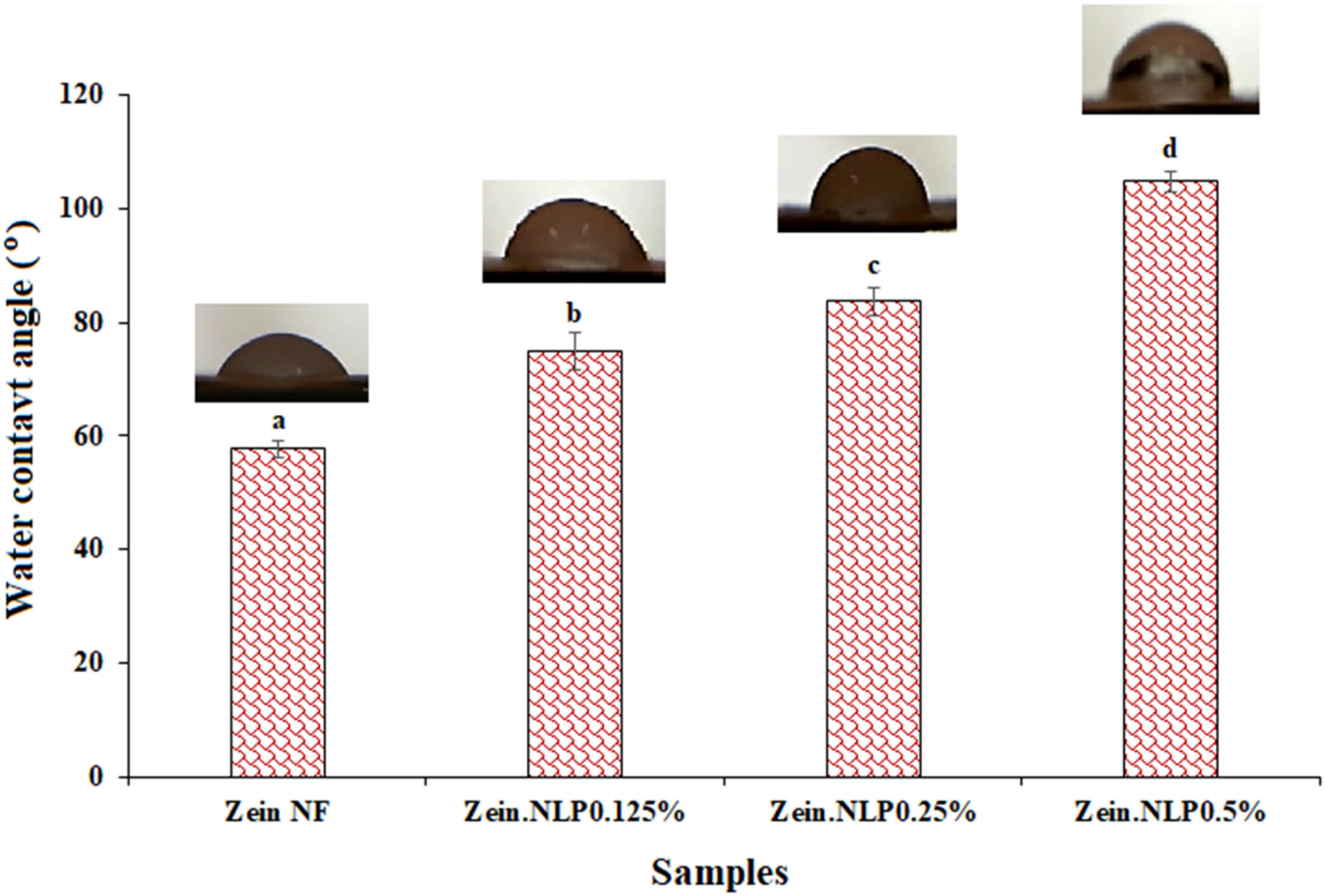
The water contact angles of neat zein, zein.NLP 0.125%, zein.NLP 0.25%, and zein.NLP 0.5% NFs. NFs, nanofibers; NLP, nanoliposome.

The water contact angle of neat zein nanofiber was 57.7°, which shows the hydrophilic property of zein NFs (Figure 7). While ethanol is employed as a substitute for water in the dissolution of zein, it is worth noting that the zein protein exhibits diminished hydrophobic characteristics as a result of the considerable quantities of hydrophilic amino acids it contains. The aforementioned observation aligns with the findings of prior research conducted on the hydrophilic characteristics of electrospun zein NFs (Amjadi, Almasi, et al., 2022a, 2022b). By adding G‐loaded NLPs in three different concentrations, the water contact angle value increased significantly (*p* < .05). In the samples containing 0.125% and 0.25% NLPs, the contact angle increased to 74.87° and 83.65°, respectively, and this value reached its maximum value in the zein.NLP0.5% sample (104.72°). These results show that by adding G‐loaded NLPs, the hydrophilic property of zein NFs was decreased and their surface became hydrophobic. Especially in the sample with the highest amount of NLPs, which has an angle greater than 90°, NFs can be considered a completely hydrophobic material. The creation of chemical connections between the active groups of lecithin, used in the production of NLPs, and the hydrophilic amino acids of zein, explains these changes. It should also be noted that according to the encapsulation efficiency of the geraniol in the present study, which was 79.23 ± 4.20%, the rest of the geraniol, which is not placed inside the NLPs, due to the hydrophobic nature of the geraniol, increases the contact angle of the NFs by increasing the number of NLPs containing the geraniol. Amjadi et al., 2020a obtained similar results regarding the effect of rosemary essential oil on the hydrophilic properties of zein/kappa (κ)‐carrageenan nanofibers. Similar results have been reported regarding the effect of natamycin (Mo et al., 2021) and perillaldehyde and thymol (Wang et al., 2022) on increasing the hydrophobicity of zein/gelatin nanofibers.

#### 3.2.6 Mechanical properties

**FIGURE 8:**
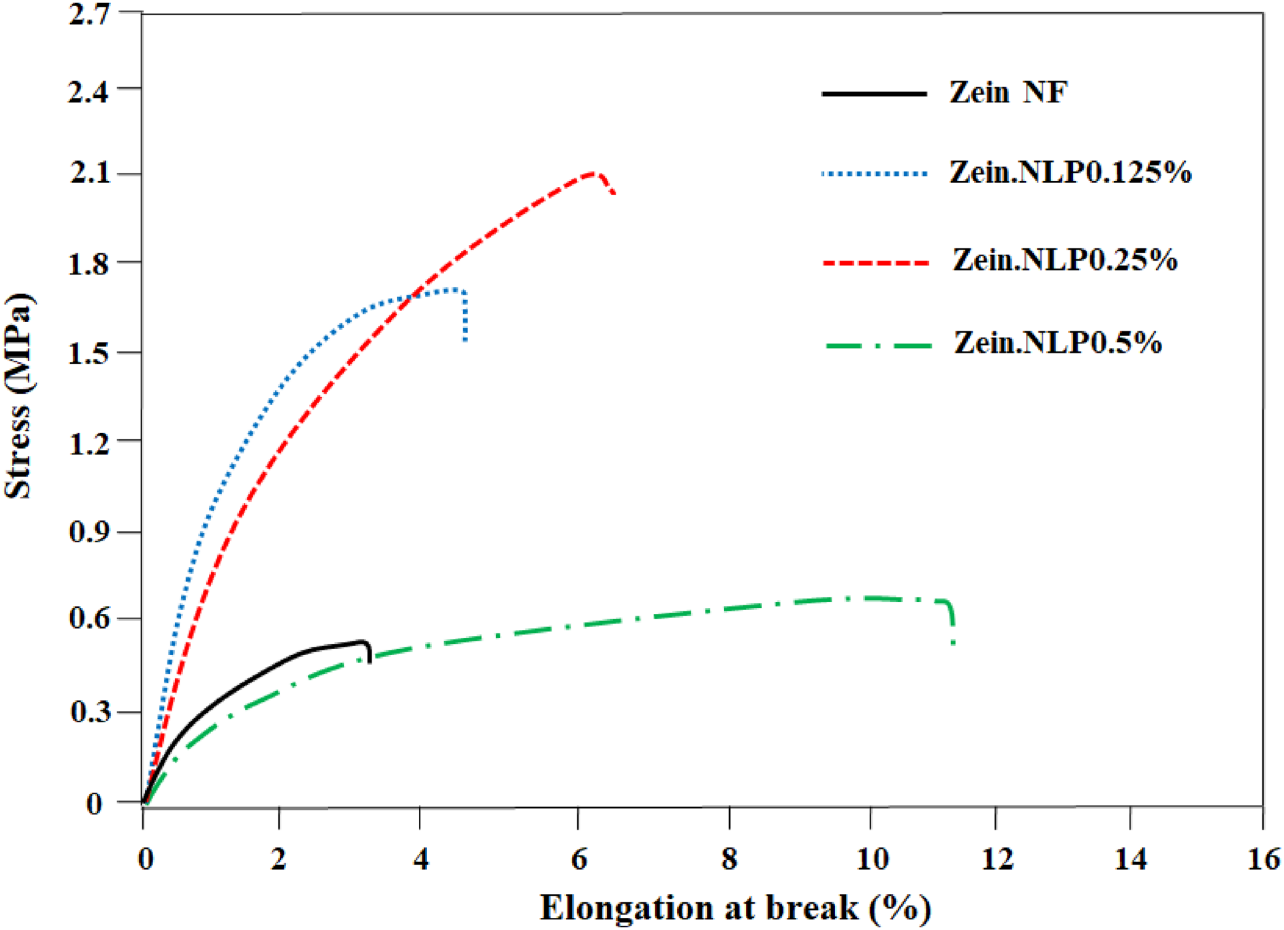
The stress–elongation curves of zein nanofibers obtained by the tensile test.

Figure 8 shows the stress–elongation curves of zein nanofibers obtained by the tensile test. Mechanical properties of the NFs, including YM, UTS, and EAB, are also provided in Table 2. As represented in Table 2, the YM, UTS, and EAB values for the neat zein were 25.95 ± 1.32 MPa, 0.54 ± 0.05 MPa, and 3.20 ± 0.54%, respectively, which have differences from the values reported by previous researchers. For example, Amjadi, Almasi, et al. (2022a) and Amjadi, Almasi, et al. (2022c) reported values of 14.92 MPa, 2.01 MPa, and 10.01% for YM, UTS, and EAB of pure zein nanofibers, respectively. Also, UTS and EAB reported for pure zein nanofibers produced in the research of Ansari et al. (2019) were equal to 8.01 MPa and 22.5%, respectively. These different and diverse values show that in addition to the type of initial material, the conditions of the electrospinning process and the characteristics of the produced material such as the diameter of NFs and the degree of entanglement will be effective on the mechanical properties of NFs. The effect of incorporating G‐loaded NLPs on the mechanical properties of NFs was found to be contingent upon the concentration of the NLPs. At concentrations of up to 0.5%, with the addition of NLPs, all three parameters YM, UTS, and EAB increased significantly (*p* > .05). This phenomenon describes the optimal effect of a substance on the physical properties of packaging components, resulting in an increase in both tensile strength and stretchability simultaneously. The formation of new chemical bonds between the hydrophilic groups of lecithin and the polar side chains of zein strands strengthens the physical structure of NFs by increasing intranetwork connections. Based on the findings of the study conducted by Cui et al. (2018), it was observed that the incorporation of liposomes containing tea tree oil into chitosan NFs resulted in an enhancement in both the mechanical strength and length of the NFs. Based on the data presented in Table 2, it can be observed that when the concentration of NLPs was increased to 0.5%, the YM reached its minimum value among all the samples. Additionally, the UTS also decreased to a degree where its difference from the control sample became insignificant (*p* > .05). The observed modifications indicate that when higher concentrations of NLPs are present, they facilitate the formation of interstrand connections between the nanocarrier and the zein. Additionally, the surplus portion of the NLPs functions as a plasticizer, impeding the establishment of the interstrand network and consequently diminishing the mechanical strength of the NFs. Amjadi et al. (2020b) also reported similar results regarding the effect of nanoliposome‐containing betanin on the mechanical properties of gelatin/chitosan nanofiber film and confirmed the softening effect of nanoliposome. The results of evaluating the mechanical properties in this research showed that the effect of G‐loaded NLPs on the mechanical properties of zein nanofibers depends on the concentration used and different effects are observed in different amounts of nanoliposome.

**TABLE 2:** Mechanical properties of neat zein, zein.NLP 0.125%, zein.NLP 0.25%, and zein.NLP 0.5% NFs.

Sample	YM (MPa)	UTS (MPa)	EAB (%)
Zein NF	25.95 ± 1.32^b^	0.54 ± 0.05^a^	3.20 ± 0.54^a^
Zein.NLP0.125%	32.55 ± 0.76^c^	1.69 ± 0.21^b^	4.18 ± 0.77^a^
Zein.NLP0.25%	41.26 ± 1.07^d^	2.01 ± 0.09^c^	6.44 ± 0.32^b^
Zein.NLP0.5%	12.87 ± 2.32^a^	0.65 ± 0.04^a^	11.54 ± 0.89^c^

*Note*: Data are expressed as mean ± standard deviation (*n* = 3) and different letters show significant differences at the 5% level in Duncan's test (*p* < .05).

Abbreviations: EAB, elongation at break; NF, nanofiber; NLP, nanoliposome; TS, tensile strength; YM, Young's modulus.

#### 3.2.7 Antimicrobial activity

Table 3 shows the antimicrobial activity of neat and G‐loaded NLPs nanofiber samples. The numerical data presented in the table indicate that the generated NFs exhibit efficacy against the four bacterial strains employed. As a result, no inhibition activity against all bacteria was detected for pure zein NFs. The previous studies also reported that neat zein‐based NFs have no antibacterial activity (Amjadi, Almasi, et al., 2022a, 2022b; Unnithan et al., 2014). The incorporation of G‐loaded NLPs in the NFs provided antibacterial activity. The observed characteristic exhibited a direct correlation with the concentration of NLPs, whereby an increase in concentration resulted in a corresponding increase in the antibacterial effect. The zein.NLP 0.5% sample exhibited the most significant impact on the four bacteria that were examined. The antimicrobial effect of geraniol has been proven in various studies (De Oliveira et al., 2018; Lira et al., 2020; Yue et al., 2017). Lira et al. (2020) attributed the mechanism of the antimicrobial effect of geraniol to the hydrophobic property of this compound. So that, geraniol can be adhered to lipids of bacterial cell membrane and interact with its components. This action causes the bacterial cell membrane to become more permeable and destroy their structures. Moreover, the antibacterial activity of active NFs against Gram‐positive bacteria (*S. aureus* and *L. monocytogenes*) was greater than its value against Gram‐negative bacteria (*E. coli* and *S. typhimurium*), which can be attributed to differences in the outer membrane structure of these bacteria. Gram‐positive bacteria have a thick cell wall composed of multiple layers of peptidoglycan. Gram‐negative bacteria, on the other hand, possess a more complex cell wall structure consisting of a thin peptidoglycan layer and an outer membrane with barrier properties. Likewise, zein NFs containing rosemary essential oil (Amjadi, Almasi, et al., 2022b), thymol (Wang et al., 2022), and cinnamaldehyde (Shao et al., 2019) have been reported to possess a similar effect on Gram‐positive and Gram‐negative microbes. These findings demonstrated that these active zein‐based NFs can be used as antimicrobial and antioxidant packaging, as well as for food preservation.

**TABLE 3:** Antimicrobial activity of neat zein, zein.NLP 0.125%, zein.NLP 0.25%, and zein.NLP 0.5% NFs.

Sample	Inhibition zone (mm)			
	*S. aureus*	*L. monocytogenes*	*E. coli*	*S. typhimurium*
Zein NF	0.0 ± 0.0^a^	0.0 ± 0.0^a^	0.0 ± 0.0^a^	0.0 ± 0.0^a^
Zein.NLP0.125%	13.5 ± 0.5^b^	11.9 ± 0.4^b^	4.4 ± 0.7^b^	6.0 ± 0.3^b^
Zein.NLP0.25%	17.6 ± 1.1^c^	13.1 ± 0.5^c^	9.5 ± 0.3^c^	7.4 ± 1.1^b^
Zein.NLP0.5%	21.8 ± 1.3^d^	19.4 ± 1.4^d^	11.2 ± 1.0^d^	10.5 ± 0.8^c^

*Note*: Data are expressed as mean ± standard deviation (*n* = 3) and different letters show significant differences at the 5% level in Duncan's test (*p* < .05).

Abbreviations: NF, nanofiber; NLP, nanoliposome.

#### 3.2.8 Antioxidant activity

**FIGURE 9:**
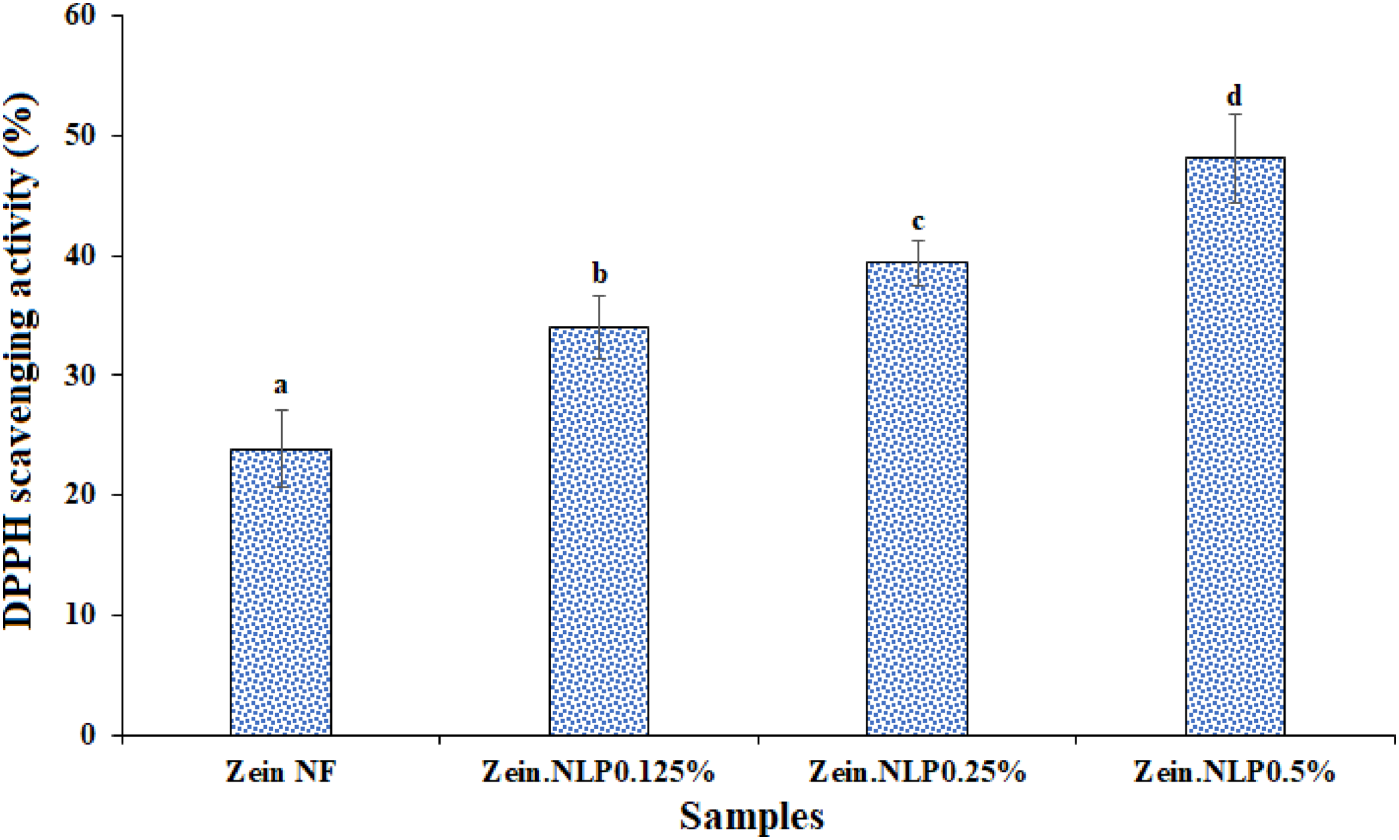
Antioxidant activity of neat zein, zein.NLP 0.125%, zein.NLP 0.25%, and zein.NLP 0.5% NFs. NF, nanofibers; NLP, nanoliposome. Different letters show significant differences at the 5% level in Duncan's test (*p* < .05).

The DPPH scavenging activity of neat zein NFs was 23.86% (Figure 9), which is consistent with earlier research (Amjadi, Almasi, et al., 2022b; Aytac et al., 2017). The existence of α‐zein in the pure zein can provide DPPH inhibition activity in zein NFs (Altan et al., 2018). Moreover, by adding the geraniol‐loaded NLPs, the antioxidant activity of NFs was significantly (*p* < .05) increased. The DPPH inhibition activity enhanced from 33.96% in zein.NLP 0.125% sample to 48.1% in the sample containing the highest concentration of NLPs. Although geraniol is mostly considered for its antimicrobial properties, its antioxidant activity has also been proven in previous studies (Kayaci et al., 2014). (Aytac et al., 2016) conducted a study on the antioxidant activity of polyvinyl alcohol NFs incorporating geraniol at concentrations ranging from 0.5% to 1%. They observed that the antioxidant activity of these NFs was found to be between 72% and 80%. Furthermore, the researchers observed that the antioxidant activity of NFs decreases by 45% following the addition of encapsulated geraniol with beta‐cyclodextrin. This finding aligns with the outcomes of the present study. In fact, by encapsulating the geraniol, the release rate of this bioactive component into the methanolic extract decreases, and as a result, the antioxidant activity is diminished compared to free geraniol‐incorporated NFs.

One of the primary objectives of encapsulation is to limit and slow down the release of the active ingredient, which reduces its functional properties in the early phases of antioxidant and antimicrobial tests. This is despite the fact that contradictory findings are also reported in the sources. Amjadi et al. (2020b) reported that the DPPH inhibition activity of samples containing betanin‐loaded NLPs was markedly greater than that of samples containing betanin‐free incorporation. They mentioned the possibility that encapsulation with NLPs could increase betanin's resistance to environmental conditions. Nevertheless, the substantial antioxidant capacity of zein NFs containing geraniol nanoliposome demonstrated that the produced NFs can be considered as active food packaging. In addition, numerous studies have demonstrated that encapsulation with NLPs and nanoemulsions increases the stability and effectiveness of antioxidant compounds for use in active films (Aziz & Almasi, 2018; Ghadetaj et al., 2018).

## 4 CONCLUSION

In this work, geraniol was successfully encapsulated with NLPs with an efficiency of 79.23%. After that, the produced NLPs were incorporated in electrospun zein NFs. The morphology of the NFs showed no significant change after the incorporation of NLPs. Due to the formation of new interactions among geraniol‐loaded NLPs and zein, improved thermal stability was observed for NFs. Moreover, the addition of G‐loaded NLPs did not adversely impact the crystalline structure of zein NFs. The results of the water contact angle measurement showed that adding G‐loaded NLPs decreased the hydrophilic property of zein NFs. The effect of incorporating G‐loaded NLPs on the mechanical parameters of NFs was found to be contingent upon the concentration of the NLPs. The incorporation of G‐loaded NLPs in the zein NFs provided antibacterial and antioxidant activities. Consequently, the zein NFs containing G‐loaded NLPs have suitable physicochemical properties and biological activity for application as an active packaging material in the food packaging industry.

## AUTHOR CONTRIBUTIONS

**Sara Gholizadeh:** Writing – original draft (equal). **Hadi Almasi:** Conceptualization (equal), Project administration (equal), Investigation (equal), Supervision (equal), Writing–reviewing and edition (equal). **Sajed Amjadi:** Formal analysis (equal); methodology (equal); project administration (equal); writing – original draft (equal). **Mehran Moradi:** Data curation (equal); investigation (equal); supervision (equal). **Nima Ghadiri Alamdari:** Data curation (equal); investigation (equal); methodology (equal). **Sorour Salmasi:** Formal analysis (equal); investigation (equal); methodology (equal); resources (equal). **Elahe Divsalar:** Investigation (equal); methodology (equal); software (equal).

## CONFLICT OF INTEREST STATEMENT

The authors declare no competing interests.

## Data Availability

Data will be made available on reasonable request.
